# Trauma research at the scene of injury – proof of concept through collaboration

**DOI:** 10.1186/1757-7241-23-S2-A3

**Published:** 2015-09-11

**Authors:** Aisling Clarkson, Nicholas C Crombie

**Affiliations:** 1 National Institute of Health Research, Surgical Reconstruction and Microbiological Research Centre, Queen Elizabeth Hospital Birmingham, UK; 2 West Midlands Ambulance Service, West Midlands, UK

## Introduction

In the United Kingdom (UK), major trauma networks became operational in April 2012. Based at Queen Elizabeth Hospital Birmingham (QEHB), supported by the Ministry of Defence and University of Birmingham, the National Institute of Health Research Surgical Reconstruction and Microbiological Research Centre (SRMRC) is one of the few 24/7 UK trauma research centres. Having demonstrated success in delivering time critical studies, collaboration with West Midlands Ambulance Service (WMAS) has resulted in transferring research to the scene of injury, demonstrated by the success of The Brain Biomarker After Trauma Study, ‘Golden-Hour.’

## Methods

Specialist WMAS staff supported by the Midlands Air Ambulance were trained for ‘Golden-Hour’ enrolling patients with a traumatic brain injury (TBI) or an Injury Severity Score (ISS)>8. Operating Procedures and consumable packs were developed allowing sampling without delaying patient care. Blood samples obtained by WMAS within one hour of injury are passed at handover to QEHB for processing. A 24/7 research team based at QEHB ensure support and sample transfer from WMAS and are responsible for consequent sampling and consenting.

## Results

Introducing a 24/7 SRMRC team into both pre-hospital and hospital settings has swiftly embedded research into routine trauma care. Whilst ‘Golden-Hour’ continues, enrolment remains ahead of predicted target with over 500 samples collected and recruitment continues to improve rapidly as WMAS staff become familiar with the protocol. The study has proved the concept allowing future collaboration and grant applications, potentially resulting in further innovations for the under-researched trauma population.

## Conclusion

Delivering research pre-hospital within one hour of injury requires a highly motivated team but is achievable. Effective integration between pre-hospital and research teams are paramount for any study to succeed. As trauma care evolves and research infrastructures within trauma networks improve, there is vast potential for further engagement with the goal of delivering evidence based pre-hospital trauma care.

